# Removal rate of 5-fluorouracil and its metabolites in patients on hemodialysis: a report of two cases of colorectal cancer patients with end-stage renal failure

**DOI:** 10.1007/s00280-023-04577-w

**Published:** 2023-08-22

**Authors:** Hirotaka Imamaki, Mitsuaki Oura, Fumiya Oguro, Yoshitaka Nishikawa, Shunsaku Nakagawa, Taro Funakoshi, Shigeki Kataoka, Takahiro Horimatsu, Atsushi Yonezawa, Takeshi Matsubara, Norihiko Watanabe, Manabu Muto, Motoko Yanagita, Yoshinao Ozaki

**Affiliations:** 1 Department of Nephrology, Hirakata Kohsai Hospital, Osaka, Japan; 2 Division of Hematology/Oncology, Kameda Medical Center, Chiba, Japan; 3 Department of Internal Medicine, Hirata Central Hospital, Fukushima, Japan; 4 Department of Health Informatics, Kyoto University School of Public Health, Kyoto, Japan; 5 Department of Therapeutic Oncology, Graduate School of Medicine, Kyoto University, Kyoto, Japan; 6 Department of Clinical Pharmacology and Therapeutics, Kyoto University Hospital, Kyoto, Japan; 7 Department of Nephrology, Kyoto University Graduate School of Medicine, Kyoto, Japan; 8 Department of Gastroenterology, Hirakata Kohsai Hospital, Osaka, Japan

**Keywords:** Hyperammonemia, 5-fluorouracil, Hemodialysis, End-stage renal disease

## Abstract

**Purpose:**

Hyperammonemia is a serious adverse effect of 5-fluorouracil (5FU) administration. Hemodialysis can be used for its management, but detailed data on the concentrations and removal rate of 5FU and its metabolites during hemodialysis remain unclear. Here, we present two cases of hemodialysis patients with end-stage renal disease who received concurrent 5FU infusion.

**Methods:**

Blood samples were collected from the hemodialysis circuit before and after the dialyzer during day 2 hemodialysis sessions, and from the internal shunt just before and after day 4 hemodialysis sessions. The serum levels of 5FU and its metabolites—α-fluoro-β-alanine (FBAL) and monofluoroacetate (FA)—were measured using liquid chromatography-tandem mass spectrometry.

**Results:**

Seven sets of blood samples were collected for case 1; the removal rates (mean ± standard deviation) of 5FU and FBAL by the dialyzer were 81.2 ± 23.2% and 96.1 ± 8.6%, respectively (*p* < 0.001). Three sets of blood samples were collected for case 2; the removal rates of 5FU and FBAL were 81.7 ± 3.9% and 94.8 ± 2.7%, respectively (*p* = 0.03). Twenty-seven sets of blood samples were collected for case 1; reductions in blood FBAL and FA levels were 49.3 ± 8.8% (*p* < 0.001) and 64.2 ± 30.3% (*p* = 0.04), respectively. Bayesian estimation yielded similar results. Three sets of blood samples were collected for case 2; reductions in the blood FBAL and FA levels were 49.9 ± 6.9% and 50.6 ± 33.0%, respectively.

**Conclusion:**

In this study, 5FU and its metabolite FBAL were directly removed from the blood by approximately 90% during hemodialysis, and the blood levels of FBAL and FA were reduced by approximately 50% with a single hemodialysis session.

**Supplementary Information:**

The online version contains supplementary material available at 10.1007/s00280-023-04577-w.

## Introduction

The overall incidence of cancer is higher in patients with end-stage renal disease (ESRD) who require renal replacement therapy, such as hemodialysis (HD), than in healthy individuals and is reported to be 4,000 per 100,000 people [1]. Fluorouracil (5FU) is a frequently used anticancer drug for gastrointestinal and other cancers [2] and for treating cancer in ESRD patients on HD [2]. Oncologic drugs account for a large proportion of drugs causing hyperammonemia [3], which is a severe but rare adverse effect of 5FU administration, especially in patients with chronic renal failure [4].

The blood levels of 5FU and its metabolites, α-fluoro-β-alanine (FBAL) and monofluoroacetate (FA), were increased before hyperammonemia in ESRD patients during treatment with 5FU [5]. FBAL is primarily excreted in the urine [6]; however, when its excretion is delayed because of impaired renal function, the body is thought to produce FA as a metabolite of FBAL [5]. FA is a potent inhibitor of the citric acid cycle, possibly leading to ammonia production [5].

To date, HD has been empirically used in patients with severe hyperammonemia due to 5FU administration [5]. However, whether dialyzers remove 5FU and its metabolites remains unclear. Therefore, the effectiveness of HD therapy remains unknown, and more information is needed on whether 5FU and its metabolites are removed by dialyzers or to what extent are 5FU metabolites, especially FA, removed from the blood during HD.

This study presents two previously unreported (new) cases of cancer patients with anuric ESRD who were receiving concurrent modified FOLFOX7 (mFOLFOX7) therapy. We measured the blood concentrations of 5FU and its metabolites before and after passing through a dialyzer during day 2 HD with continuous 5FU administration to determine the quantity of 5FU and its metabolites removed directly from the blood by the dialyzer in HD. We also measured the concentration of 5FU and its metabolites in peripheral venous blood before and after HD on day 4 after completion of continuous 5FU administration to examine the extent to which blood levels of 5FU metabolites change before and after dialysis.

## Case presentation

### Case 1

A 74-year-old man (height 158 cm, weight 50 kg) with diabetic nephropathy as the primary disease started HD three times a week in March 2018. Due to ascending colon cancer and lung metastasis (pT3N0M1, stage IV, adenocarcinoma [tub1], RAS mutant, BRAF wild type [WT]), he underwent right hemisection in 2019. He started mFOLFOX7 therapy in March 2019.

Since September 2019, oxaliplatin was discontinued due to grade 2 peripheral neuropathy according to Common Terminology Criteria for Adverse Events version 5.0 (CTCAE), while 5FU/leucovorin was continued. The 5FU dose was 1600–1200 mg/m^2^ (66–50%) every 2 weeks. Despite dose reduction to 2000 mg/m^2^ in the first cycle to mitigate the risk, hyperammonemia occurred. Therefore, the 5FU dose was 1600 mg/m^2^ for the next four cycles (cycles 2–5) and was further reduced to 1200 mg/m^2^ in cycles 6–28 because of CTCAE grade 3 diarrhea in cycle 5. In October 2020, 5FU was finally discontinued due to disease progression.

The dialyzer material was polysulfone. The Supplement Data show the other dialysis conditions.

### Case 2

A 73-year-old woman (height 154 cm, weight 50 kg) with diabetic nephropathy as the primary disease was first diagnosed with rectal cancer with liver metastasis in 2018. She underwent Hartmann surgery in January 2019 (pT3N0M1, stage IV, adenocarcinoma [tub2], RAS mutant, BRAF WT), and started leucovorin simple therapy with 5FU in March 2019. In January 2020, irinotecan therapy was started as a second-line treatment; however, the disease worsened. She started HD therapy in March 2020 and mFOLFOX7 therapy in May 2020. After dialysis induction, as the patient insisted on continuing chemotherapy, the dose was reduced accordingly. 5FU/leucovorin was well tolerated, and mFOLFOX7 containing oxaliplatin, which had never been used before, was administered for a total of 6 cycles at intervals but was discontinued due to disease progression. The dialyzer material was polymethylmethacrylate. The Supplement Data show the other dialysis conditions.

## Materials and methods

Both patients were hemodialyzed three times a week via an internal shunt (Supplement Fig. 1). 5FU was administered continuously for 46 h every Tuesday (day 1). Dialysis on day 2 (Wednesday) was performed concurrently with 5FU administration. Blood samples were obtained from the dialysis circuit before (pre-dialyzer) and after (post-dialyzer) passing through the dialyzer at the middle of the dialysis while 5FU was being administered **(**Analysis 1; Supplement Fig. 2).

**Fig. 1:**
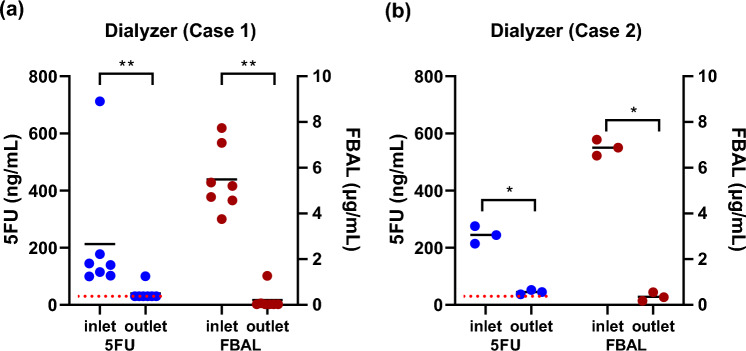
**a**, **b** Concentrations of 5FU and FBAL in a dialyzer inlet/outlet blood sample (day 2). Daugirdas Kt/V = 1.518 (Patient 1), Daugirdas Kt/V = 1.20 (Patient 2). Each horizontal rule represents the average value; red dashed lines indicate the detection limits. P-values are calculated with the Peto & Peto modification of the Gehan–Wilcoxon test using the NADA package on R (*p < 0.05, **p < 0.01). *5FU* 5-fluorouracil, *FBAL* alpha-fluoro-beta-alanine, *FA* fluoromonoacetate, *HD* hemodialysis

**Fig. 2:**
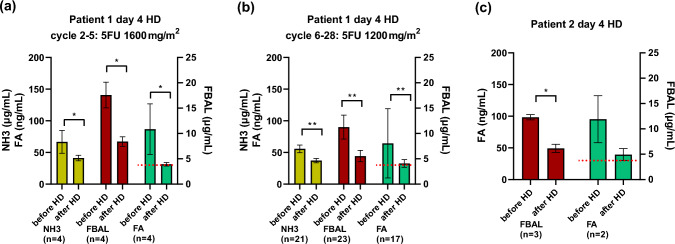
**a**–**c** Concentrations of NH3, FBAL, and FA in a venous blood sample before/after HD (day 4). Each bar represents the average ± standard deviation; red dashed lines indicate the detection limits. *p*-values are calculated with the Peto & Peto modification of the Gehan–Wilcoxon test using the NADA package on R (*p < 0.05, **p < 0.01). *5FU* 5-fluorouracil, *FBAL* alpha-fluoro-beta-alanine, *FA* fluoromonoacetate, *HD* hemodialysis

During dialysis on day 4 (Friday), after 46 h of continuous 5FU administration, blood was drawn on the arterial needle side of the internal shunt just before the start and at the end of dialysis (Analysis 2; Supplement Fig. 3). The removal rate on day 2 was calculated using the following formula: (pre-dialyzer serum concentration – post-dialyzer serum concentration)/pre-dialyzer serum. The removal rate on day 4 was calculated using the following formula: ([pre-hemodialysis serum concentration – post-hemodialysis serum concentration]/pre-hemodialysis serum concentration) ´ 100. The dialysis efficiency (KT/V sp) was calculated using the Daugirdas equation [7].

### Blood collection, storage, and measurements

Blood samples were collected after receiving written consent from the patients. Then, blood was centrifuged at 3500 rpm for 5 min with 3490 g (times gravity) centrifugal force, and the supernatant was frozen at – 30 °C.

The ammonia (NH_3_) concentration was determined using the DRI-CHEM SLIDE NH3-WII (normal range, 12–66 μg/dL; Fujifilm, Tokyo, Japan). 5FU, FBAL, and FA concentrations were determined using liquid chromatography-tandem mass spectrometry with LCMS-8040 (Shimadzu, Kyoto, Japan) with a detection limit of 30 ng/mL [8].

### Statistical analysis

All analyses were performed using R, version 4.1.1 (https://www.R-project.org/). The mean values, differences, and removal rates were calculated; *p*-values and 95% confidence intervals (CIs) were calculated using the Wilcoxon rank sum test when there were no non-detects (values below the detection limit). When there were non-detects, we assumed the values using three methods. First, non-detects were assumed to be 30 ng/mL unless otherwise stated, and we calculated the mean values, differences, and removal rates. Second, p-values and 95% CIs were calculated using the Peto and Peto modification of the Gehan–Wilcoxon test with the NADA R package (version 1.6–1.1) [8]. Lastly, Bayesian estimation was performed using the RStan package (version 2.21.2). In Bayesian estimation, the test values were assumed to follow a normal distribution and to be positive. Means, differences, and removal rates were computed using the Hamiltonian Monte Carlo algorithm. All Bayesian estimation results were confirmed to be convergent with an R-hat of 1.0. Data are presented as mean ± standard deviation unless indicated otherwise.

## Results

Blood samples were drawn during regular dialysis, and no adverse events associated with sample collection were observed. The results were nearly identical and reproducible in both cases; malignancy was controlled during chemotherapy administration, and side effects were within acceptable limits.

### Analysis 1: pre-dialyzer and post-dialyzer concentrations during continuous 5FU administration (day 2)

#### Case 1

A total of seven measurements were taken. Dialysis efficiency was 1.52 ± 0.12. The serum 5FU concentration was high only in the first measurement: 712.27 ng/mL in the pre-dialyzer blood sample and 100.51 ng/mL in the post-dialyzer blood sample. The actual 5FU concentration pre- and post-dialyzer was measured only for the first measurement. The removal rate was 85.9%. After the second measurement, the post-dialyzer 5FU concentration was lower than the limit (≤ 30 ng/mL). The median pre-dialyzer value was 139.45 ng/mL. Assuming a serum concentration of 30 ng/mL of 5FU in the second through seventh post-dialysis serum samples, the removal rate was 77.3 ± 6.1% in seven measurements (p < 0.001, Fig. 1a). The removal rate by Bayesian estimation was 77.4% (95% CI 28.5–97.5%).

For FBAL, as in the case of 5FU, the first blood concentration measurement was the highest but was not significantly different in the subsequent measurements. In five of the seven measurements, the post-dialyzer blood concentrations were lower than the detection limit. The FBAL removal rate for the two times that actual blood concentrations could be measured before and after dialysis was 91.0 ± 10.5%. Assuming that the concentration was five times below the sensitivity value (0.03 μg/mL), the mean of the seven measurements yielded pre- and post-dialyzer blood FBAL concentrations of 5.49 ± 1.43 (median, 5.20) and 0.21 ± 0.47 μg/mL, respectively, with a removal rate of 97.0 ± 5.9% (*p* < 0.001, Fig. 1a). Bayesian estimation of the seven measurements revealed a removal rate of 85.9% (95% CI 46.4–98.7%). Blood FA concentrations were lower than the limit for all seven measurements.

#### Case 2

A total of three measurements were performed. The mean dialysis efficiency was 1.23 ± 0.15.

The pre- and post-dialyzer 5FU blood levels were 244.7 ± 30.6 (median, 244.3) and 44.7 ± 7.7 (median, 45.2) ng/mL, respectively. In all three measurements, the blood 5FU and FBAL levels pre- and post-dialysis exceeded the sensitivity value of the measurement. The removal rate with the dialyzer was 81.6 ± 3.0% (*p* = 0.03, Fig. 1b). Bayesian estimation was not performed, as there were no censored values below the lower limit.

The pre- and post-dialyzer FBAL blood concentrations were 6.88 ± 0.35 and 0.36 ± 0.19 μg/mL, respectively. The removal rate by the dialyzer was 94.8 ± 2.6% (*p* = 0.03, Fig. 1b). Bayesian estimation was not performed, as there were no censored values below the lower limit. Blood FA levels were below the detection limit for all three measurements.

### Analysis 2: changes in blood levels of 5FU metabolites by HD (day 4)

#### Case 1

The FBAL and FA concentrations were measured 27 times (cycles 2–28). The mean dialysis efficiency of these 27 sessions was 1.54 ± 0.15. For FBAL. The pre- and post-dialyzer blood concentrations were 12.2 ± 3.3 (median, 11.4) and 6.0 ± 1.5 (median, 5.5) μg/mL, respectively. The removal rate by dialysis was 50.1 ± 9.0% (*p* < 0.001). The removal rates were 51.8 ± 5.3% (*p* = 0.01) and 49.8 ± 9.5% (*p* < 0.001) in cycles 2–5 and 6–28, respectively (Fig. 2a, b). Bayesian estimation was not performed, as there were no censored values.

FA levels were measured 5 of 22 times in pre- and post-dialyzer blood samples. The pre- and post-dialyzer blood concentrations were 112.0 ± 92.1 (median, 71.9) and 40.1 ± 7.7 (median, 38.1) ng/mL, respectively. The removal rate by dialysis was 49.0 ± 26.0% (*p* = 0.04). For FA, 16 of 27 occasions included a non-detect either in a pre-dialyzer or post-dialyzer sample. Six of 27 occasions included non-detects in pre-dialyzer and post-dialyzer samples.

When non-detects were assumed as 30 ng/mL and six sessions with non-detects were excluded, the removal rates were 57.8 ± 17.7% (*p* = 0.01) and 37.7 ± 18.6% (*p* < 0.001) in cycles 2–5 and cycles 6–28, respectively (Fig. 2a, b). According to Bayesian estimation, the removal rates were 69.3% (95% CI 36.7–91.7%) and 64.3% (95% CI 26.4–92.0%) in cycles 2–5 and 6–28, respectively.

#### Case 2

FBAL and FA concentrations were measured thrice; one of the three times, the pre- and post-dialyzer FA values were non-detects. The dialysis efficiency of these three times was 1.23 ± 0.16.

serum FBAL concentration at the beginning and end of dialysis was 12.3 ± 0.5 (median, 12.1) and 6.2 ± 0.8 (median, 5.8) μg/mL, respectively. FBAL removal rate by dialysis was 50.1 ± 4.3% (*p* = 0.03, Fig. 2c). Bayesian estimation was not performed, as there were no censored values below the sensitivity value.

For FA, when taking the mean values of two measurements without non-detects, the blood concentrations at the beginning and end of dialysis were 95.3 ± 37.0 and 39.5 ± 9.6 ng/mL, respectively. The removal rate was 57.3 ± 6.5% (*p* = 0.1, Fig. 2c). Bayesian estimation was not performed because of the small number of valid values.

## Discussion

To our knowledge, this is the first study to report that approximately ≥ 90% of 5FU and FBAL can be removed directly from the blood by dialysis and that a single HD session can reduce blood FA levels by at least 50%.

FA is extremely toxic and difficult to study in vivo, and its removal rate by dialysis has not been reported.

Dialysis improves 5FU-induced hyperammonemia [5, 9, 10]. However, since the metabolic pathway includes hepatic metabolism [11], few studies have examined whether dialysis truly removes the causative agent from the body, and none have examined the quantitative removal rate. Our data also suggest that HD is effective in managing or preventing hyperammonemia during 5FU administration. Rengelshousen et al. [10] calculated the dialysis clearance of FBAL to be approximately 73–84%, which was comparable to our direct measurement of the FBAL removal rate by dialysis.

Although not proven, 5FU-induced hyperammonemia may be caused by NH_3_ overproduction during 5FU metabolism and the reduced ability of the body to process NH_3_. With normal renal function, approximately 60–90% of the administered 5FU is excreted in the urine within 24 h, 10% as unchanged 5FU and 70% as FBAL [6, 9]. Over 80% of 5FU is metabolized by dihydropyrimidine dehydrogenase, which is primarily found in the liver, with FBAL as an end product [11].　The remaining 5FU is ultimately metabolized to carbon dioxide and NH_3_ and broken down in the urea cycle. When FBAL excretion is delayed due to renal dysfunction, blood FA levels increase [5]; FA specifically inhibits aconitase, a coenzyme in the citric acid cycle [11]. Thus, the conversion of citric acid to isocitrate is reduced and the citric acid cycle is inhibited. This in turn inhibits the urea cycle—a metabolic pathway for NH_3_—which may decrease NH_3_ metabolism causing hyperammonemia [11].

In case 1, a polysulfone membrane dialyzer was used, which is the most widely used membrane material, excellently removes small proteins and β2-MG from small molecules, suppresses complement activation, and is very biocompatible [12]. In case 2, polymethylmethacrylate membrane was used, which has cytokine adsorption, anti-inflammatory, and antioxidant properties [13]. However, we found no studies examining the relationship between dialyzers and 5FU, FBAL, or FA concentrations.

Three important factors define substance removal efficiency in dialysis: molecular weight, protein binding rate, and volume of distribution. If the molecular weight of a substance is < 500 Da, it is removed by the dialyzer [14]. The molecular weight of 5FU and FBAL is 130 Da and 107 Da, respectively, which explains the high substance removal percentage. Additionally, dialysis membranes are designed to keep out albumin, a blood protein with a molecular weight of approximately 60,000 Da, and substances bound to albumin in the blood are less likely to be removed during HD. The protein binding rate of 5FU is 0–4% [8, 15], substantiating the high removal rate by dialyzers. The protein binding rates of FBAL and FA are unknown. Herein, > 90% of FBAL was removed from the blood during dialysis, suggesting that the protein binding rate of FBAL is low; on day 4, the blood FBAL and FA levels were reduced by half after one HD session, suggesting that the protein binding rate of FA is not high. The average volume of FBAL distribution for capecitabine, a prodrug of 5FU, was previously estimated to be 73.6 L [16]. Thus, its tissue transfer rate is presumably low; hence, it is easily removed by dialysis. However, the distribution volume of FA remains unknown.

This study had some limitations. First, tissue FBAL and FA concentrations were unknown. Second, for dihydropyrimidine dehydrogenase, the enzyme that catalyzes 5FU degradation, activity assessment is not currently available to the public, even on a commercial basis, in our country. Therefore, we were unable to assess it.

In conclusion, our study shows that HD can help in treating or preventing hyperammmonemia, a potentially serious side effect of anticancer therapy with 5FU in patients with ESRD. Future studies are needed to determine the extent of FA removal by the dialyzer.

### Supplementary Information

Below is the link to the electronic supplementary material.

Supplementary file1 (PDF 308 KB)

Supplementary file2 (PDF 199 KB)

Supplementary file3 (PDF 199 KB)

Supplementary file4 (DOCX 21 KB)

## Data Availability

The data that support the findings of this study are available from the corresponding author upon reasonable request.
