# Supplementary material for: Removal rate of 5-fluorouracil and its metabolites in patients on hemodialysis: a report of two cases of colorectal cancer patients with end-stage renal failure
Source: Cancer Chemother Pharmacol. 2023 Aug 22;93(2):161–7. doi: 10.1007/s00280-023-04577-w (PMC10853355; doi:10.1007/s00280-023-04577-w)
Supplement: Supplementary file 1 — Supplementary file1 (PDF 308 KB) [file 280_2023_4577_MOESM1_ESM.pdf]

**Supplement Fig. 1** Sample collection process – (1) Terminology

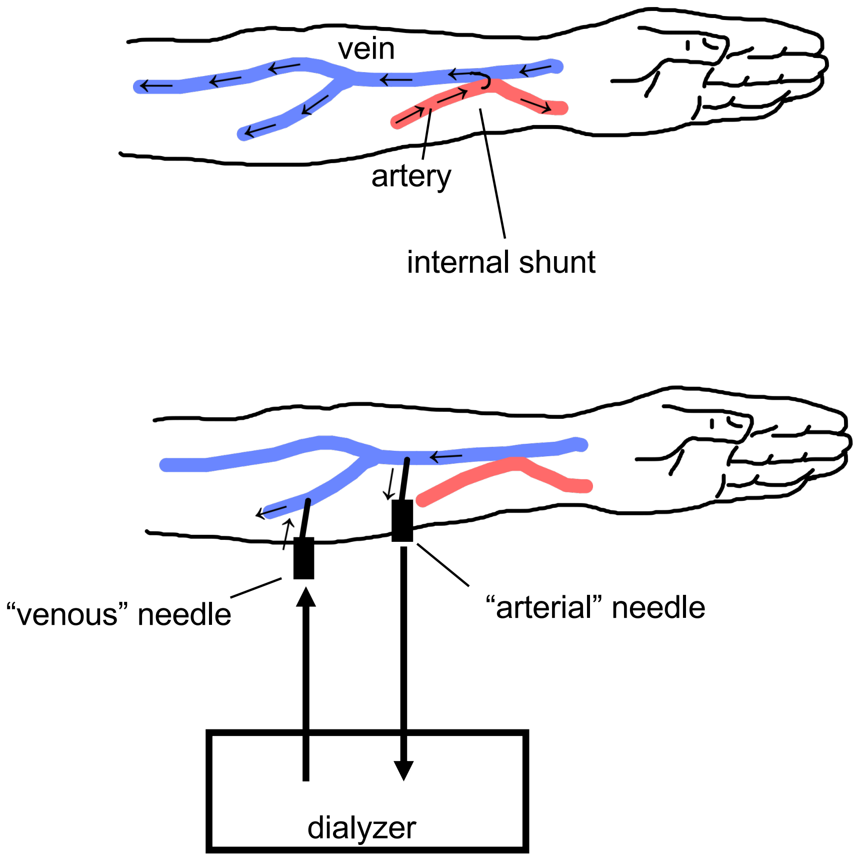


Article title: Removal rate of 5-fluorouracil and its metabolites in patients on hemodialysis: A report of two cases of colorectal cancer patients with end-stage renal failure
Journal name: *Cancer Chemotherapy and Pharmacology*
Author names: Hirotaka Imamaki, Mitsuaki Oura, Fumiya Oguro, Yoshitaka Nishikawa, Shunsaku Nakagawa, Taro Funakoshi, Shigeki Kataoka, Takahiro Horimatsu, Atsushi Yonezawa, Takeshi Matsubara, Norihiko Watanabe, Manabu Muto, Motoko Yanagita, and Yoshinao Ozaki
Affiliation and e-mail address of the corresponding author: Hirotaka Imamaki, Department of Nephrology, Hirakata Kohsai Hospital, Osaka, Japan; hiroimamaki2022@gmail.com
