# Supplementary material for: Removal rate of 5-fluorouracil and its metabolites in patients on hemodialysis: a report of two cases of colorectal cancer patients with end-stage renal failure
Source: Cancer Chemother Pharmacol. 2023 Aug 22;93(2):161–7. doi: 10.1007/s00280-023-04577-w (PMC10853355; doi:10.1007/s00280-023-04577-w)
Supplement: Supplementary file 4 — Supplementary file4 (DOCX 21 KB) [file 280_2023_4577_MOESM4_ESM.docx]

**Supplementary Information**

**Title: Removal rate of 5-fluorouracil and its metabolites in patients on hemodialysis: A report of two cases of colorectal cancer patients with end-stage renal failure**

**Journal name:** Cancer Chemotherapy and Pharmacology

**Authors:**

Hirotaka Imamaki^1^, Mitsuaki Oura^2^, Fumiya Oguro^3^, Yoshitaka Nishikawa^4,5^, Shunsaku Nakagawa^6^, Taro Funakoshi^5^, Shigeki Kataoka^5^, Takahiro Horimatsu^5^, Atsushi Yonezawa^6^, Takeshi Matsubara^7^, Norihiko Watanabe^8^, Manabu Muto^5^, Motoko Yanagita^7^, and Yoshinao Ozaki^8^

**Affiliations**

^1^ Department of Nephrology, Hirakata Kohsai Hospital, Osaka, Japan

^2^ Division of Hematology/Oncology, Kameda Medical Center, Chiba, Japan

^3^ Department of Internal Medicine, Hirata Central Hospital, Fukushima, Japan

^4^ Department of Health Informatics, Kyoto University School of Public Health, Kyoto, Japan

^5^ Department of Therapeutic Oncology, Graduate School of Medicine, Kyoto University, Kyoto, Japan

^6^ Department of Clinical Pharmacology and Therapeutics, Kyoto University Hospital, Kyoto, Japan

^7^ Department of Nephrology, Kyoto University Graduate School of Medicine, Kyoto, Japan

^8^ Department of Gastroenterology, Hirakata Kohsai Hospital, Osaka, Japan

**Corresponding author**

Hirotaka Imamaki

E-mail: [hiroimamaki2022@gmail.com](mailto:hiroimamaki2022@gmail.com)

**Supplement Data: Dialysis Conditions**

Case 1

Dialysis time, 3 or 3.5 hours

Blood flow rate, 200 mL/min

Dialysate volume, 500 mL/min

Dialyzer material, polysulfone

Dialyzer name, APS-25SA (Asahi Kasei Medical, Inc.)

Case 2

Dialysis time, 4 hours

Blood flow rate, 200 mL/min

Dialysate volume, 500 mL/min

Dialyzer material, polymethylmethacrylate

Dialyzer name, BG-1.6PQS (Toray Medical Co., Ltd.)
